# Rituals in Death and Dying: Modern Medical Technologies Enter the Fray

**DOI:** 10.5041/RMMJ.10182

**Published:** 2015-01-29

**Authors:** Michael Gordon

**Affiliations:** Professor of Medicine, Medical Program Director, Palliative Care, Baycrest Health Sciences, University of Toronto, Toronto, Canada

**Keywords:** Cross-cultural death, death, dying, rituals

## Abstract

In the absence of immortality, the human species has over the millennia developed rites and rituals to help in the passing of life to honor the person who is dying or has died or in some way demonstrate their “courage” and perseverance as well as duty even in the face of almost certain death. The centuries-old traditions of the gathering of loved ones, the chanting of prayers, the ritual religious blessings are in the process of being replaced by the “miracles” of modern medical technology.

## INTRODUCTION

“I find getting older a lousy deal,” the actor Woody Allen explained. “There is no advantage in getting older. I’m 74 now, and you don’t get smarter, wiser, more mellow or kindly. Your back hurts more; your eyesight isn’t as good. It’s a bad business. I advise you to avoid it if you can. It’s better to be younger and get the girl.”^1^ When asked how he felt about death, Allen deadpanned, “My relationship to death remains the same. I’m strongly against it.”^2^ Although using blackish humor, Allen’s representation of death and dying reflects what many deeply feel.

Throughout history, the human species has had to deal with the reality of death. There were those in our historical past who formulated death as part of the religious construct of the universe. All religions and cultures have their rituals, prayers, and beliefs related to the process of dying and what those who are left behind by the death of someone who matters were supposed to do. During the dying period or in anticipation of expected death, all cultures, societies, and religions had—and continue to have—a range of rituals that allow those left alive to anticipate, adjust to, and in some ways honor the person who is dying and also afterwards when death has occurred. Much has been written on the subject, and depending on where one is practicing medicine some issues may be more common or more challenging than others.^3^

For the purposes of this article’s exploration, the focus will be on Western primarily monotheistic faiths as it would be impossible to cover the world of varying religious beliefs and practices. Moreover, for much of the Western world, at present many of the end-of-life challenges and conflicts about which a literature exists are for those who either have no primary over-riding religious belief or are from the monotheistic faiths, all of which historically derive from the Old Testament with major variations that have occurred throughout Western history.

With the progress of modern medicine, it appears that some of the long-standing rituals surrounding impending death and dying may have become modified or at least influenced by what appears to be the prospects of changing the trajectory of dying through the advent of modern medical advances. These interventions include salvage medications and technological innovations such as cardiac pacemakers, respiratory support, antibiotics to treat those infections that in the past portended death in those in the later stages of various diseases (as Sir William Osler memorably stated, “Pneumonia is the old man’s friend”^4^), and, in the extreme, attempts at cardiopulmonary resuscitation (CPR).

In the last-mentioned case the intervention is relatively recent in the history of contemporary medicine. The first contemporary reports about the potential benefits of closed chest cardiac massage associated with respiratory support were published in the early 1960s.^5^ From then on there was a massive and rapid adoption of this technique especially in the United States and then gradually in other Western jurisdictions. The techniques gradually improved, the results in different populations were enhanced, and the acceptance of this somewhat extreme intervention became part of the mainstream of medical practice.

The use of CPR became so pervasive despite often rather dismal outcomes in elderly patients at the extremes of life, or in those with medical conditions that could no longer be effectively treated, that in the early 1980s, with the advent of the concept of autonomy in medical decision-making, individuals were formally asked if they wanted to pursue CPR as part of their attempt to thwart the effect of a cardiac arrest. For many individuals and family members this option was interpreted as a “return from the dead” or the avoidance of otherwise certain death. The popular media have been instrumental in promoting that view of CPR, and the exaggerated benefits have appeared to become incorporated into the common lay understanding of its use in medical care—literally at all ages and in all situations.^6^ This is often true of other medical treatments in the last period of life such as antibiotics for end-of-life infections. Those infections, primarily respiratory and urosepsis, are often part and parcel of the body’s inability to mount a response to infection which frequently ends up being the final common pathway to dying; attempts made at thwarting the infection with the use of intravenous antibiotics are quite often “against all odds” of clinical success. In one report on terminal stage treatment of those with hematological disorders similar to the situation in the case described below, 90% of patients received antibiotics during the last week of life.^7^

## CASE STUDY

An 84-year-old male with a more than 10-year history of chronic myeloid leukemia developed a blast transformation during the eleventh year of his disease. He underwent chemotherapy which eventually lost its beneficial effect. This resulted in frequent episodes of anemia and then bleeding from thrombocytopenia and recurrent pulmonary tract and urinary tract infections, the last-mentioned being aggravated by prostatic hypertrophy with periods of retention.

With his and his family’s concurrence he was treated on an agreed-to regimen of blood products including packed cells and platelets, with the latter assisting to decrease his episodes of bleeding which were always disconcerting to the patient and family. He was cared for at home with the assistance of a very devoted family and was also given opiates for pain especially bone pain, which was complicated by serious arthritis which he had for years—with the medication providing reasonable relief of symptoms.

As his condition deteriorated he required periodic admission to hospital to provide the antibiotics and blood products under supervision and to provide opiates for serious physical discomfort. The family had been spoken to by the treating hematologist and indicated that they had already decided, not without significant soul-searching and family and physician-directed discussions, against any resuscitation should his “heart stop.” They wanted to continue with the current treatments despite accepting the principles of palliative care but would not forgo the blood product and antibiotic treatments. During the few days prior to his death, which was without significant discomfort, he continued to get parenteral fluids, as he had ceased eating and drinking, and was given broad-spectrum cephalosporin antibiotics which the physicians described to the wife and involved children as “very powerful” or at times “the most powerful” medications.

After he died, at the *shiva*^8^ the wife lauded the doctors and the hospital for their excellent care and said to all who asked about the last days of life that, “he received the best and most powerful antibiotics that were available and got blood products almost until the very end.” In keeping with her own sense of her rightful devotion to her husband, it was clear that she was able to emphasize the fact that he was comfortable until the end and that “everything that could be done was done.” It was clear from listening to her retell this story over and over again just how important it was that she could frame his death in those terms, literally and symbolically, focusing on each of the extreme medical treatments that he received.

## INCORPORATION INTO THE CONTEMPORARY RITUALS OF DYING

In the Western world there is now a very complex mixture of cultures and religions that carry with them age-old traditions melded with the influence that modern medicine has to offer which may impact on what might have been traditional, cultural, and religious behaviors commonly practiced. A modification of the expectations anticipated from the health care system usually evolves in keeping with the offerings and standards of practice in their adopted country. Any medical practitioner in North America or Western Europe would likely have experienced such a phenomenon.

What does this mean for the traditional narrative about the normally expected and adhered-to rituals of death and dying? For example, in the three major monotheistic Abrahamic religions the idea of the obligation of the family and therefore the physicians and other health care providers to “save a life” is a profound obligation and one that is completely transformed in jurisdictions that are replete with modern technologies that might be used in order to achieve this goal, although virtually impossible other than for mere moments, hours, or sometimes days.

A recent court case in Ontario concerns the case of a patient from a country that clearly would not have been able to undertake the requested prolonged treatment in an intensive care unit (ICU) due to his being in a minimally conscious state for a year. The family persisted in their request to continue his treatment despite the opinions expressed universally by the physicians treating him that he was not going to recover. Among the arguments that were used to move the court to order the continuation of his treatment primarily were the issues surrounding the proper interpretation of consent to treatment and many legal nuances related to Ontario law and the issue of what treatment might constitute a new treatment for which consent would be necessary.^8^ Very low down in the Supreme Court decision, reference was made to his religious values which were interpreted by his family as requiring all attempts to maintain his life to be pursued even in the face of overwhelming odds and mounting costs.

The Western ethical concept of distributive justice, which could not ignore his financial drain on the health care system, did not even enter in any substantial way into the final legal decision which in essence obligated the physicians to continue to treat him; his religious and cultural norms, with the rituals that accompany them, seemed to be deemed pre-eminent within his family and in many of the commentaries related to the final Court decision.^9^ Although the Supreme Court did not focus primarily on the religious aspect of the patient and wife’s religious views and preferences, the media, including the ethics and religious-based media, interpreted the decision as favoring and acknowledging the importance of religious views and preferences in such decision-making. As noted in a commentary in the Rotman Institute of Philosophy, “A proper analysis of harms and benefits must take into consideration the values of the patient because harms include violations of the patient’s autonomy, sense of self and core beliefs. It is conceivable that a patient may value continued life over physical discomfort, as is the case with Mr. Rasouli whose religious commitments require him to preserve his life even in the face of great suffering.”^10^

## COMMONLY PRACTICED RITES AND RITUALS PRIOR TO DEATH

As explained in her article on cultures and the rituals and rites of dying and death, O’Gorman gives an example from within Judaism: “Orthodox Jewish rituals begin as death draws near and the dying person and family take part in farewell rites. The dying asks forgiveness for their past errors and expresses hope for the welfare of the survivors. The family says final goodbyes and recites together prayers of affirmation and hope. As soon as death has occurred the body is prepared for burial by ‘chevra kadisha’ a holy society made up of specially trained lay volunteers.”^8^,^11^ Other important components of the rituals of Jewish dying and death have a profound meaning for those who ascribe to the tenets of Judaism and for many who throughout their lives were not particularly attentive to the rituals; they, too, often follow the ancient and almost universal practices that are part of their Jewish narrative.^8^ For a Catholic, as is explored in an article on Hispanic last rites and rituals, there is the almost universal ritual whereby, “A Catholic, on his or her deathbed, is given last rites by a priest and is anointed with holy oil for this purpose. The priest hears the dying person’s confession and offers absolution. The patient, when able, receives Communion and a blessing from the priest.”^12^

## CONTEMPORARY MEDICAL PRACTICE

If one works in a teaching medical center the opportunity is there to observe how young physicians in particular frame the questions they ask family members who may be acting in the role of substitute decision-makers (SDMs) or, in other jurisdictions, health care proxies or surrogates. A common approach to the CPR dialogue may start with the question, “What do you want us to do if your mother/father’s heart stops beating suddenly? Should we try to start it again through CPR or *just* let him/her die?” When you think about the construct of a question framed this way it is very difficult for the person making the decision to give a deliberated answer unless it had been discussed previously with the person on whose behalf the decision is being made. For the decision-maker it would appear that there is a real life-and-death choice between almost (if not completely) futile CPR and the goal of a comfortable more dignified death. It would be unusual for the question to be framed as the more accurate but clearly less acceptable, “if your mother’s heart stops as part of her dying process, do you want us to attempt first of all to shock it electrically and then pound on her chest in a way that may very well break a few ribs (as she is very frail), with the very unlikely prospect that we may restart the heart? Even if that is accomplished, she will most likely die soon after as really her time has come. The alternative would be to make her comfortable during this period and do our best to avoid any discomfort or suffering.”

The response to the first framing of the option of CPR leaves the family thinking that they might be making a decision that will determine whether their beloved mother will live or die. One often hears family members say, “I cannot feel that I was responsible for my mother’s death” or “how could I be asked in essence to kill my mother?” The other commonly used misnomer which adds to the general misconception surrounding CPR is when a family asks for a “full code.” There is no meaningful concept of a partial code—either it is a “code” (which means “full”) or in essence no code. For even the most meagre potential for beneficial resuscitation to occur the undertaking must be a “full code.” Cardiopulmonary resuscitation is often deemed to be the default position—that is to say, in the absence of a Do Not Resuscitate (DNR) order, CPR would be applied unless it is clear clinically that it would be contraindicated; this position usually depends on two criteria: that it is witnessed and that it is unexpected.

The request for a “full code” would be the equivalent within the ethical decision-making framework of, rather than choosing between viable options of treatment presented by the medically responsible person, the decision-maker requesting a treatment even when there is no clinical evidence that any benefit will be derived from it. Physicians in general have the ethical duty and responsibility not to undertake treatments that they believe have no potential for therapeutic benefit or may in fact cause one of many harms—physical, psychological, or, for that matter, distributive in the way that they utilize health care resources. We do not generally intubate and provide respiratory support for individuals who are clearly dying for the sake of a family wanting to get “every last breath” out of them. At times it appears to border on assault rather than treatment.^13^

The same request can be made for end-of-life antibiotic treatment, even though in many circumstances it is well understood by the treating physicians that a course of antibiotics (for what is in essence, at that point in the trajectory of the disease process, the final overwhelming infection that often either accompanies or causes death) has little to do with therapeutic benefit. Although in some cases there may be a short reprieve with the use of antibiotics in such circumstances, the final infection usually follows soon after as the *coup de grâce*.^14^,^15^

Although not as dramatic as the *ritual* of CPR, the fact that the treatment of an ultimately fatal infection does often take some time fulfills the concept of the ritualistic aspect that the treatment has (the intravenous bag, the change of medication bottles, the taking of pulse, blood pressure, and temperature, the ringing of bells and alerting alarms)—a *rite* of passage more akin to praying, chanting, and singing of hymns to help the dying pass into the next world. Also how the physician frames the discussion may lead the family to only one acceptable course—to undertake antibiotic treatment: “If your mother gets an infection like pneumonia” do you want us to try and treat it with antibiotics? Just imagine a dutiful child rejecting such an offer for treatment when common knowledge includes that pneumonia in the usual sense is usually successfully treated with antibiotics—why not my mother who may be dying, but not quite yet. And yet even within the context of a setting for which death is the expected outcome, end-stage antibiotics are often administered almost ritually.^14^,^15^

## THE RITUAL ASPECT OF END-OF-LIFE MEDICAL INTERVENTIONS

With this brave new world of remarkable medical technologies and the readily available and popularized knowledge of its availability, it is not surprising that ordinary people when acting as substitutes for their loved ones naturally want “everything” done that modern medicine can provide. But more than that natural desire is the place of such interventions within the concept of the rituals of dying. Whereas in the past a family might gather around the bed of a dying person who more than likely would be dying at home, it is now much more common that the person is a patient in an acute care system although they may be a resident or patient in a long-term care facility. Within the acute care setting for sure, the expectation that “everything” will be done to prolong life is often a given assumption, unless conversations have occurred prior to the illness and some modicum of advance care planning has occurred so that previously expressed wishes about rejecting interventions such as CPR result in a documented DNR order to avoid any such last-minute salvage intervention.

In the absence of such advance planning and clearly documented verbal or written expression of wishes, it becomes important for the family to feel confident that, when the end actually comes, they can say to themselves and then to all that ask, often at the ceremonies and practices that usually occur to acknowledge the death of the person: “everything that could be done was done.” One often hears family members describing the last period of life noting in great detail all the things that were done, at their behest, to “save” their loved one: they used the “most powerful antibiotic possible, they tried to bring him back to life by shocking him four times—they would not give up.” This becomes part of the ritual narrative of dying, and by allowing it to happen the family can be comfortable that they fulfilled their filial duty by doing what in the contemporary world is expected and available as part of the process of dying: not chants and candles and prayers, but beeps, flashing lights, screaming of medication orders, and the magical “shocking” with resuscitation paddles, which has its own drama to this new ritualistic process. This awareness is gradually making its way into contemporary medical and other health-care-related literature, and the physician’s awareness of the differences and nuances of culture and belief is becoming more emphasized in educational forums.^16^

## SUGGESTIONS: HOW TO AVOID THE USE OF MARGINALLY EFFECTIVE MODERN MEDICAL TECHNOLOGY IN END-OF-LIFE SITUATIONS

While rites and rituals traditionally and historically used by many cultures and religions have been replaced with modern technologies, steps can be taken to avoid the often disappointing results of such interventions to help families have peace of mind after death occurs in a loved one. Among the many steps now being explored by many medical and health care organizations to avoid excessive use of inappropriate medical technologies and interventions in end-of-life situations, such undertakings under the umbrella term of *advance care planning* are becoming very popular. Educational undertakings and having early conversations with one’s family members seem to be promoted currently with the hope that proper understanding and knowledge, and time to ponder and give one’s deep-felt opinions prior to crisis situations, might avoid the use of treatments which may in fact prolong suffering without any chance of major benefit. However, for those who hold deep-seated religious beliefs that may be interpreted as requiring all necessary medical interventions to be attempted, the use of advance care planning may not succeed in protecting individuals from the rites and rituals of end-of-life modern technology.^17^

## CONCLUSION

Throughout human history, rites and rituals at the end of life have been important components of all societies, cultures, and religions. Until the advent of modern medicine with all its complex technologies, these activities were primarily focused on what family members and designated religious or cultural leaders brought into the process to assist and guide the dying person and his family and community through the dying process and through many defined activities to celebrate the person’s life after death had occurred. Modern medicine has in many ways replaced the personal rites and rituals, the songs, chants, music, and appeal to the guiding spirits with complex medical interventions. These are often the happenings that are remembered and referred to during the after-death “celebration” of the person’s life and last days, hours, and moments of death. Physicians and other health care professionals must become more aware—through participating in advance care planning and discussion of their patients’ important values, and listening carefully to family comments during the dying period—because what may seem to them to be just part of clinical medicine can, for the family of the dying person, have a very profound and lasting effect on how they recall and recount that last and very important period of life and prologue to death.

## Abbreviations:

CPR: cardiopulmonary resuscitation;
DNR: do not resuscitate;
SDMs: substitute decision-makers.
